# Identification of endogenous control genes for normalisation of real-time quantitative PCR data in colorectal cancer

**DOI:** 10.1186/1471-2199-11-12

**Published:** 2010-02-01

**Authors:** Elrasheid AH Kheirelseid, Kah Hoong Chang, John Newell, Michael J Kerin, Nicola Miller

**Affiliations:** 1 Department of Surgery, National University of Ireland, Galway, Ireland; 2 Biostatistics Unit, Clinical Research Facility, National University of Ireland, Galway, Ireland

## Abstract

**Background:**

Gene expression analysis has many applications in cancer diagnosis, prognosis and therapeutic care. Relative quantification is the most widely adopted approach whereby quantification of gene expression is normalised relative to an endogenously expressed control (EC) gene. Central to the reliable determination of gene expression is the choice of control gene. The purpose of this study was to evaluate a panel of candidate EC genes from which to identify the most stably expressed gene(s) to normalise RQ-PCR data derived from primary colorectal cancer tissue.

**Results:**

The expression of thirteen candidate EC genes: *B2M, HPRT, GAPDH, ACTB, PPIA, HCRT, SLC25A23, DTX3, APOC4, RTDR1, KRTAP12-3, CHRNB4 *and *MRPL19 *were analysed in a cohort of 64 colorectal tumours and tumour associated normal specimens. *CXCL12, FABP1, MUC2 *and *PDCD4 *genes were chosen as target genes against which a comparison of the effect of each EC gene on gene expression could be determined. Data analysis using descriptive statistics, geNorm, NormFinder and qBasePlus indicated significant difference in variances between candidate EC genes. We determined that two genes were required for optimal normalisation and identified *B2M *and *PPIA *as the most stably expressed and reliable EC genes.

**Conclusion:**

This study identified that the combination of two EC genes (*B2M *and *PPIA*) more accurately normalised RQ-PCR data in colorectal tissue. Although these control genes might not be optimal for use in other cancer studies, the approach described herein could serve as a template for the identification of valid ECs in other cancer types.

## Background

Colorectal cancer (CRC) is one of the most common causes of cancer worldwide affecting almost a million people annually and resulting in approximately 500,000 deaths [1]. Approximately 5% of individuals born today will be diagnosed with colorectal cancer during their lives, representing a lifetime risk of 1 in 19. CRC remains a serious threat to life with approximately 20% of patients presenting with late stage metastatic disease. Although 5 year survival rates are favourable at 80-90% for early stage disease, this drops significantly to less than 10% with the presence of distal metastasis.

The majority of colorectal tumours originate from adenomatous precursor lesions and develop along a well-defined adenoma-carcinoma sequence. According to this model the culmination of mutational events including activation of oncogenes and loss of function of tumour suppressor genes results in the emergence of carcinomas [2]. Molecular profiling across the spectrum of normal-adenoma-tumour tissue types has yielded many candidate genes in the search for novel molecular diagnostic and prognostic markers and treatment strategies [3-5]. In latter years real-time quantitative (RQ-) PCR has become established as the gold standard for accurate, sensitive and rapid quantification of gene expression [6,7]. In comparison to alternative methods such as Northern blotting and Ribonuclease Protection Assays (RPA), RQ-PCR has been universally adopted as the transcriptomic method of choice due to its superiority with regard to speed, sensitivity, reproducibility and the wide range of instrumentation and reagents commercially available.

To accurately quantify an mRNA target by RQ-PCR, samples are assayed during the exponential phase of the PCR reaction during which the amount of target is assumed to double with each cycle of PCR without bias due to limiting reagents. Analysis of cycle threshold (C_t_), the cycle number at which signals are detected above background, can be used to estimate gene expression levels by relating C_t _values either to a standard curve (absolute quantification) or to a control gene (relative quantification). The latter method requires the generation of standard curves of known copy number for each target and so is limited due to logistical issues associated with the generation of standards in studies of multiple gene targets. Relative quantification is the most widely adopted approach and as the name suggests, quantification of gene expression is based on the analysis of a target gene whose expression is normalised relative to the expression of a control gene. Central to the reliable determination of gene expression is the choice of control gene with which to normalise real-time data from target genes. Normalisation can be achieved using endogenous or exogenous controls; however the use of endogenous control (EC) genes is the most widely adopted approach as it excludes variation associated with differences in amounts of template RNA. Vandestompele *et al *2002 described a normalisation method whereby geometrical averaging of multiple EC genes improved accuracy [8]. This approach has been adopted to reliably measure levels of gene expression in many studies in different tissue types including breast [9-11], lung [12], kidney [13], brain [14] and liver [15].

An ideal EC gene (or genes) should be stably expressed and unaffected by parameters such as disease status and in the case of CRC, should remain unaffected by whether a tissue was derived from normal, adenoma or carcinoma lesions. Traditionally *GAPDH *(glyceraldehyde phosphate dehydrogenase) has been widely used to normalise RQ-PCR data. A common feature of earlier studies was that the stability of reference gene expression between different sample types was assumed with little consideration paid to validation of these EC genes as suitable normalisers. More recent studies have brought into question the stability of commonly used EC genes such as *GAPDH *on the basis that gene expression levels have been found to vary in response to treatment or as a result of physiological, pathological or experimental changes. For example, alteration in oxygen tension and hypoxia were found to be associated with wide variation in *GAPDH, B-ACTIN *and *CYCLOPHILIN *expression [16]. In addition, *GAPDH *expression was found to be strongly unregulated in diabetic patients and down-regulated in response to the administration of bisphosphonate compounds in the treatment of metastatic breast cancer [17]. Other evidence indicates that neoplastic growth can affect EC expression levels [18]. Goidin *et al *[19] found differences in the expression of *GAPDH *and *B-ACTIN *in two sub-populations of melanoma cells derived from a tumour in a single patient. Treatment agents such as dexamethasone, deprenyl and isatin also affect EC gene expression [20,21]. Schmittgen *et al *[22] reported increased expression of *GAPDH, B2M, 18S rRNA *and *β-ACTIN *in fibroblasts after the addition of serum: evidence of the effect of experimental conditions on EC expression. These findings were further supported by Wu *et al *[23] in their investigation of the effect of different skin irritants on *GAPDH *and PolyA+ RNA expression. *GAPDH *was found to be involved in age-induced apoptosis in mature cerebellar cells [24] and also as a tRNA binding protein present in the nuclei of HeLa cells [25].

As the use of unreliable ECs can result in inaccurate results, the identification of the most reliable gene or set of genes at the outset of an investigation is critical. Thus far, a pervasive stably expressed gene (or genes) has yet to be identified across all tissue types [26,27]. This would indicate that the identification of robust ECs at the outset of transcriptomic analysis would yield more reliable and meaningful RQ-PCR data.

The aim of this study was to evaluate a panel of thirteen candidate EC genes from which to identify the most stably expressed gene (or genes) to normalise RQ-PCR data derived from primary colorectal tumour and tumour associated normal (TAN) tissue. Six of the candidate EC genes were selected from the literature and represent the most frequently studied reference genes in cancer including, but not limited to, colorectal cancer. Each gene was previously reported as being constitutively expressed in various tissues. These EC genes included *B2M (beta-2-microglogulin) *[*5*], *HPRT (hypoxanthine guanine phosphoribosyl transferase 1) *[*3*,*28*], *GAPDH *[*29*], *ACTB (beta-actin) *[*30*], *PPIA (peptidyl-prolyl isomerise A) *[*9*] and *MRPL19 (mitochondrial ribosomal protein L19) *[*9*]. The remaining seven genes included *HCRT, SLC25A23, DTX3, APOC4, RTDR1, KRTAP12-3, and CHRNB4*. The latter candidates were selected from an unpublished whole genome microarray dataset of 20 human tumour specimens and represented the most stably expressed probes with a fold-change of 1.0-1.2, (p < 0.05). Expression of *CXCL12 *[31], *FABP1 *[32], *MUC2 *[33] and *PDCD4 *genes were chosen as targets against which to measure the effects of candidate EC expression on the basis of their previously identified roles in tumourigenesis. In addition to its tumour suppressor properties, *PDCD4 *[34] also has diagnostic and prognostic utility and represents a promising target for anti-cancer therapy.

## Results

### Range of Expression of Candidate EC Genes

A range of C_t _values was observed across the candidate EC genes in tumour and TAN tissue from CRC patients as indicated in table 1. Only samples with a standard deviation < 0.3 from the mean C_t _of the triplicates were included for further analysis. The expression of *RTDR1, HCRT, APOC4 *and *KRTAP12-3 *could not be determined in all 64 tissue samples, resultantly these candidates were excluded from further analysis.

**Table 1:** Clinico-pathological patient data for tumour and tumour associated normal colorectal tissues

Clinicopathological Variable	Number of Patients N = 42
**Gender**	
Males	29
Females	13

**Mean Age (SD)**	66.5 (12.84)

**Tumour Location**	
Colon	12
Rectum	30

**Tumour Diameter (mm)**	
<10	11
10-15	15
>15	17

**Tumour Thickness (mm)**	
<30	15
30-40	12
>40	15

**Distant Metastasis**	
M0	36
M1	6

**Nodal Status**	
N0	22
N1	11
N2	9

**UICC Stage**	
Stage 0	6
Stage I	10
Stage II	10
Stage III	11
Stage IV	5

**Tumour Differentiation**	
Well	12
Moderate	24
Poor	6

**Mucin Secretion**	
Mucinous	8
Non-mucinous	34

Mean C_t _values for the remaining genes ranged from 19.48 (± 0.14 s.e.m) for *B2M *to 32.30 (± 0.19 s.e.m) for *CHRNB4. B2M *displayed the narrowest range of C_t _values between 17.5 and 21.5 (mean 19.5 ± 0.14 s.e.m, range of 4.04) followed by *PPIA *and *MRPL19*, while *ACTB *had the widest range of C_t _values between 33.8 and 21.1. The genes broadly fell into three categories, those least abundant genes with mean C_t _values of 27-32 (*SLC25A23, MRPL19, DTX3 *and *CHRNB4*), moderately abundant genes with mean C_t _values of 22-26 (*HPRT *and *ACTB*) and the most abundant highly expressed genes with mean C_t _values of 19-21 (*B2M, PPIA *and *GAPDH*) Table 2.

**Table 2:** Cycle threshold (Ct) values of candidate EC genes and *PCDC4 *in colorectal tissues

EC Gene	C_t _Range	C_t _Min	C_t _Max	Mean C_t _± s.e.m	Standard deviation (SD)
B2M	4.03	17.47	21.51	19.48 ± 0.14	1.04

PPIA	4.13	17.78	21.91	19.90 ± 0.14	1.06

GAPDH	5.80	18.51	24.32	21.00 ± 0.17	1.29

ACTB	12.74	21.08	33.32	25.14 ± 0.34	2.61

HPRT	8.54	22.74	31.28	26.68 ± 0.25	1.89

DTX3	6.6	24.95	31.56	28.62 ±0.17	1.37

SLC25A23	7.26	24.48	31.74	27.36 ±0.19	1.54

CHRNB4	9.40	27.99	37.38	32.30 ±0.19	2.15

RTDR1	-	30.59	UD	35.82 ±0.36	2.15

HCRT	-	33.96	UD	38.46 ±0.29	1.67

APOC4	-	UD	UD	-	-

KRTAP12-3	-	33.16	UD	36.95 ±0.19	1.46

MRPL19	4.10	26.70	30.80	28.62 ± 0.13	0.98

CXCL12	13.54	21.85	35.39	25.77 ± 0.32	2.61

FABP1	15.57	16.61	32.19	20.83 ± 0.40	3.24

MUC2	17.71	17.62	35.33	22.43 ± 0.53	4.16

PDCD4	11.92	21.35	33.27	24.56 ± 0.32	2.59

*B2M *and *PPIA *were the most abundantly expressed genes, having the lowest mean Ct values while *MRPL19 *was the least abundantly expressed gene with average Ct values > 26. Both *B2M *and *PPIA *had the lowest range in their Ct values.

### Identification of Optimal EC genes

**Figure 1:**
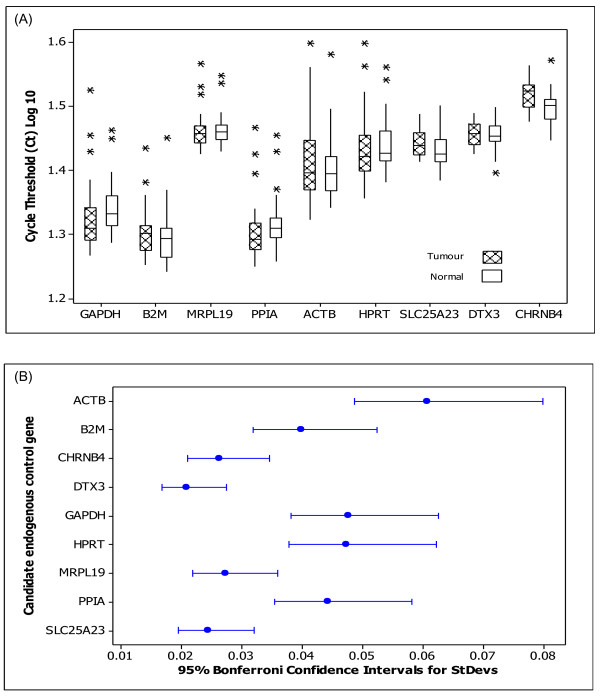
**Scaled expression levels and variation of each candidate EC gene**. (A) Log 10 of cycle threshold of candidate EC genes *ACTB, B2M, GAPDH, HPRT, MRPL19, SLC25A23, DTX3, CHRNB4 *and *PPIA *in tumour and normal colorectal tissues. Boxplot shows interquartile range box, median, range whiskers and outliers (*). Within gene, differences were found in expression between tissue groups in both *SLC25A23 *(p = 0.040) and *CHRNB4 *(p = 0.002) but not the other genes (p > 0.05) (ANOVA). (B) Variation associated with EC gene expression. There was a significant difference in variation associated with gene expression (p < 0.001) with *ACTB, GAPDH *and *HPRT *showing greater variation than *B2M, MRPL19 *or *PPIA. DTX3, CHRNB4 *and *SLC25A23 *showed the least variations (Levene's test).

**Figure 2:**
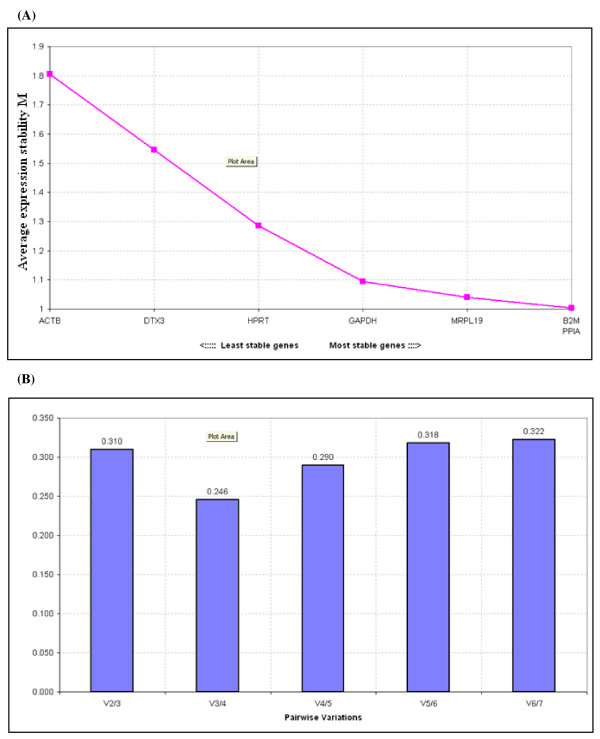
**Analysis of candidate EC genes using geNorm**. **(A): Average expression stability values of eligible EC genes**. Expression stability of the control genes as calculated by geNorm. Stability value M is based on the average pair-wise variation between all genes. The least stable gene with highest M value was excluded and M value recalculated till end up with the most stable pair. **(B): Determination of optimal number of control genes for normalisation**. The GeNorm programme calculates a normalisation factor (NF) which is used to determine the optimal number of EC genes required for accurate normalisation. This factor is calculated using the variable V as the pairwise variation (Vn/Vn + 1) between two sequential NFs (NFn and NFn + 1). To meet the recommended cut off V-value which is the point at which it is unnecessary to include additional genes in a normalisation strategy. The recommended limit for V value is 0.15 but it is not always achievable. In this instance, the GeNorm output file indicated that the optimal number of genes required for normalisation was three.

Scaled expression levels across the remaining nine candidate ECs analysed (figure 1) indicated within-gene differences in expression between tumour and normal tissue groups in both *SLC25A23 *(p = 0.040) and *CHRNB4 *(P = 0.002) but not in the remaining genes (p > 0.05), (figure 2A). Therefore, *SLC25A23 *and *CHRNB4 *genes were excluded from further analysis. Significant differences in variance of EC expression were identified using Levene's test (p < 0.001, figure 2B). These findings necessitated further evaluation of each candidate EC gene prior to their possible use to accurately quantitate gene expression levels of the target genes *CXCL12, FABP1, MUC2 *and *PDCD4*.

**Figure 3:**
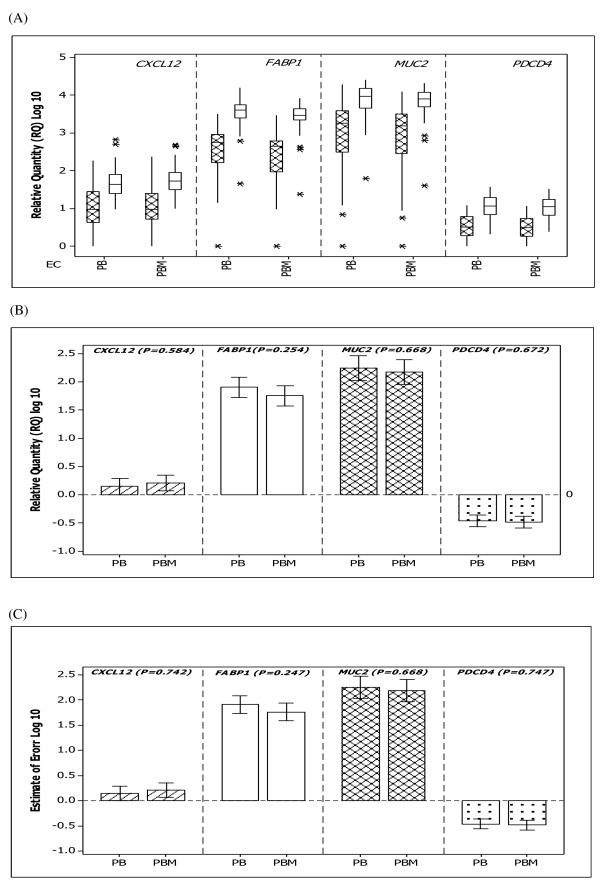
**Relative quantity of *CXCL12, FABP1, MUC2 *and *PDCD4 *in colorectal tissue**. Error bars indicate 95% confidence intervals. No significant differences in the relative quantities of target genes were found using a combination of *PPIA *and *B2M *(PB) genes in comparison to the use of combination of *PPIA, B2M *and *MRPL19 *(PBM) EC genes (ANOVA).

**Figure 4:**
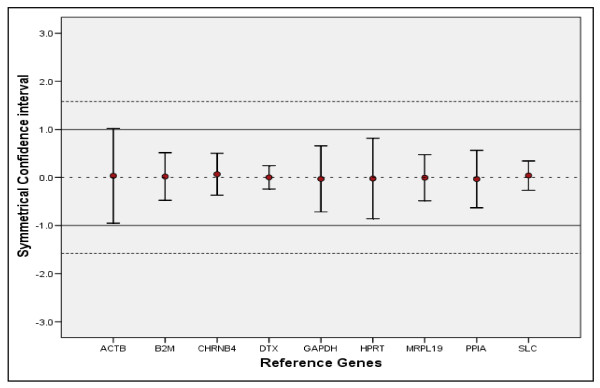
**Equivalence test for candidate control genes in colorectal tissue**. Differences in logarithmic expression levels between tumour and normal tissues (●) are indicated. The upper and lower bars of each line indicate the upper and lower limits of the symmetrical confidence intervals, respectively. The deviation area (-1, 1) for a fold change of 2 or less is plotted as a continuous line while the deviation area of (-1.58, 1.58) for a fold change of 3 is plotted as a dotted line.

The stability of candidate EC genes was analysed using geNorm [8] and NormFinder [35] programmes. Stability was further evaluated using qBasePlus [8,36], a commercially available RQ-PCR data mining package. These programmes were used to calculate amplification efficiency-corrected relative quantities from raw fluorescence data. The ranking of candidate EC genes as determined by each of these programmes is illustrated in Table 3. In the case of GeNorm the variable V indicating the pairwise variation (Vn/Vn+1) between two sequential normalisation factors (NFn/NFn+1) indicated that three EC genes was the optimal number of genes for accurate normalisation (figure 2), however, target genes expression did not differ significantly if two rather than three EC genes were used (figure 3). Use of all three programmes confirmed that *B2M *and *PPIA *was the best combination of genes for normalising RQ-PCR data in CRC tissues (table 3). The Equivalence test [37] was used to examine the expression of candidate ECs. All genes were equivalently expressed between the normal and tumour colorectal tissues using a fold cut-off of 2 (figure 4).

**Table 3:** Ranking and best combination of EC genes as determined by geNorm, NormFinder and qBasePlus.

Rank	GeNorm		NormFinder		qBaseplus	
	
	Gene	Stability (M)	Gene	Stability (M)	Gene	CV value
1	GAPDH	1.477	MRPL19	0.008	GAPDH	0.555

2	MRPL19	1.467	B2M	0.015	PPIA	0.659

3	PPIA	1.535	HPRT	0.016	HPRT	0.775

4	B2M	1.636	PPIA	0.017	MRPL19	0.914

5	HPRT	1.813	GAPDH	0.018	B2M	0.923

6	DTX3	2.251	DTX3	0.020	ACTB	0.957

7	ACTB	2.454	ACTB	0.026	DTX3	5.829

Best Combination	B2M/PPIA	1.005	B2M/PPIA	0.007	B2M/PPIA	0.460

For GeNorm, lower stability values (M) indicate greater stability. In the case of NormFinder, stability is calculated from inter- and intra-group variation. By grouping the tissues into tumour and normal the best combination of genes was identified. For geNorm stability was based on the estimation of pair-wise variation. QBasePlus through its components, geNorm and qBase, identified coefficient of variation (CV) and stability (M) values and thereby the best combination of genes for normalisation only when more than one gene is used.

### Association between EC genes and target genes

**Figure 5:**
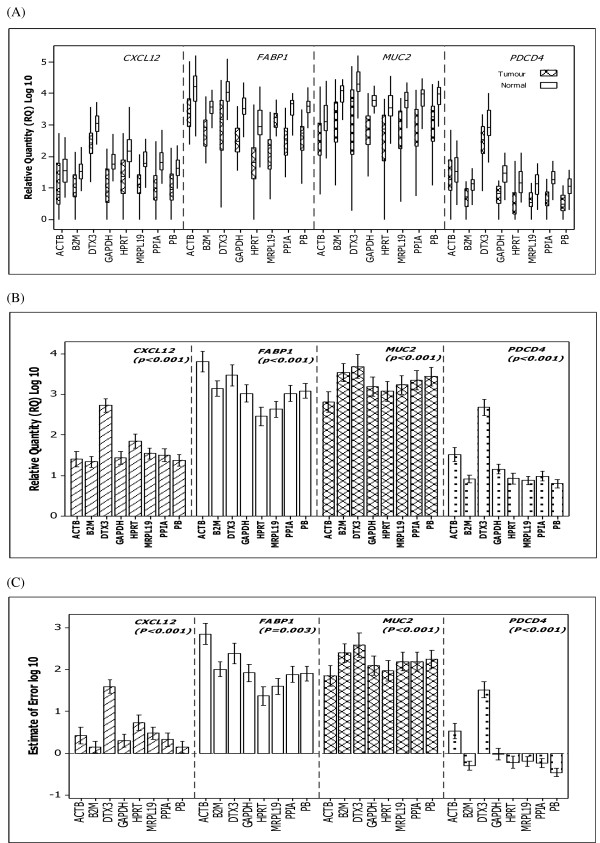
**Relative quantity of target gene expression in colorectal tissues relative to each EC gene and to the geometric mean of the combined use of *PPIA *and *B2M *(PB)**. (A) Target gene expression in tumour versus normal using either individual candidate EC genes or the PB combination. (B) Significant differences in relative gene expression values as determined using ANOVA to compare mean expression levels across all tissues using either individual EC genes or PB in combination. (C) One way ANOVA indicating a reduction in the magnitude of error when the PB combination was used to normalise expression of *CXCL12 *(p < 0.001) and *PDCD4 *(p < 0.001) in comparison to the use of individual EC genes. See Table 1 Additional files for Post Hoc tests. Error bars indicate 95% confidence intervals.

There was a significant effect of the expression of the candidate EC genes on relative expression of *CXCL12 *(p < 0.001), *FABP1 *(p < 0.001), *MUC2 *(p < 0.001) and *PDCD4 *(p < 0.001) (figure 5A and 5B). Moreover, a significant effect of the choice of EC with regard to the estimation of error (figure 5C) was also detected. These findings were further confirmed for each EC gene compared to each other by ANOVA Tukey post hoc tests (Additional files 1). The combined use of *B2M *and *PPIA *significantly reduced the magnitude of error in comparison to the use of either gene individually for both *CXCL12 *and *PDCD4 *expression. The addition of a third EC gene (*MRPL19*) to the *B2M*/*PPIA *combination did not result in any further improvement of the estimation of error (figure 3C).

### Non-normalised expression levels of target genes

**Figure 6:**
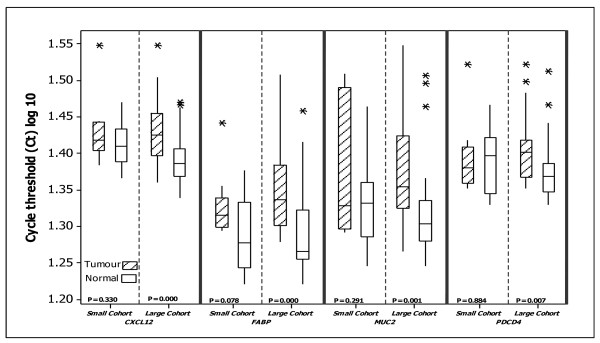
**Non-normalised cycle threshold (C_t_) of *CXCL12, FABP1, MUC2 *and *PDCD4 *in colorectal tissue**. Using this approach, the expression of each gene appears to be down-regulated in tumours compared to normal tissues in the large cohort of patients (30 tumour and 34 normal tissue specimens), similar to previous published reports of reduced expression in colorectal tumours. No significant differences were noted in expression levels of target genes when using the small cohort of patients (10 tumour and 10 normal tissue specimens) (2-sample t-test). This confirms the effect of sample size on findings when using non-normalised C_t _values and therefore the importance of normalisation especially in such type of studies

To assess whether normalisation was necessary in a large cohort such as this in which the biological effect of the target genes is already established, we compared the expression of the four target genes in tumour *vs*. normal tissues using non-normalised cycle threshold (C_t_) values in the entire sample set (n = 64) and in a sub-set of randomly selected 10 normal and 10 tumour tissues (n = 20). This analysis showed down-regulated target gene (*CXCL12, FABP1, MUC2 *and *PDCD4*) expression in tumour compared to normal tissues (figure 6), in keeping with their documented tumour suppressor functions, when using the larger set of samples. The unchanged target gene expression levels in the large cohort could be explained by the fact that in larger sample sizes the biological milieu may diminish subtle variations in individual samples. In contrast, when the smaller sample size was used, no significant differences in target gene expression were observed. Furthermore the expression levels of *PDCD4 *appeared slightly higher in tumours than in normal tissues. When the same subset of 20 samples were normalised with *PPIA/B2M*, significant differences in target gene expression were observed.

## Discussion

Since its introduction in 1996 [38] many methods have been developed for the analysis real-time quantitative PCR data. Relative quantification has come to the fore as the method of choice due to its superior flexibility and reduction in inherent variation associated with sample preparation. Prior to the availability of high-throughput realtime PCR instrumentation, a handful of genes were commonly used to normalise real-time data. Major technological advances enabling high throughput analysis of both samples and target genes have enabled investigation and validation of putative EC genes prior to their use to normalise target gene expression. It is now accepted that the use of more than one gene to normalise RQ data improves experimental accuracy compared to the use of a single EC gene [8,35,38]

In their study of EC gene expression in breast and colon cancer tissues Tricarico *et al *[39] illustrated significant variation in the expression levels of 10 commonly used housekeeping genes including 18S rRNA, both between individuals and between biopsies taken from the same patient. They concluded that normalisation to a single EC gene was inappropriate for human tissue samples. Moreover, Vandesompele *et al *identified errors of up to 6.5 fold when a single EC gene was used in comparison to the use of multiple genes for data normalisation [8] thereby clearly indicating the potential for superior accuracy when due consideration is paid to the choice of EC genes.

Many analytical programmes for relative quantification have been developed, certain of which enable the identification of EC genes from a study population [37,40,41]. In the present study the stability of expression of candidate EC genes was determined using a pair-wise comparison model: geNorm [8] and an MS Excel ANOVA based model, NormFinder [35]. No effect of disease status EC gene expression was identified in colorectal tissue. Since both geNorm and NormFinder are based on the assumption that candidate genes are not differentially expressed between samples, this was an important first step prior to their continued use [9,10].

In this study GeNorm was used to identify the most stably expressed EC genes from our panel of candidates and also provided a measure of the optimal number of EC genes. *B2M *and *PPIA *were identified as the most stable pairing. In order to achieve a pair-wise variation value (V) below the cut-off of 0.15 additional genes should theoretically be used; however this cut-off point is not absolute [14] and may not always be achievable [42]. No significant difference in target gene expression was observed when the top three most stable EC genes identified by geNorm were used confirming that using of a pair of genes may be more practicable given cost, work load and sample availability considerations.

NormFinder was designed to identify EC genes with the lowest stability values; these values are calculated based on intra- and inter-group variation. In this study NormFinder was used to define the best combination of genes using tumour and normal as group identifiers in the calculations. *MRPL19 *was selected as the most stable gene using these criteria; however *B2M *and *PPIA *were highlighted as the best combination of genes with even lower stability value compared to *MRPL19 *alone. QBasePlus real-time PCR data manager programme was developed based on geNorm and qBase [36] algorithms. QBasePlus was used to confirm our selection of the *B2M *and *PPIA *pairing as the best combination of ECs in colorectal tissue.

Equivalence testing was developed in biostatistics to address the situation where the aim is not to show the difference between groups, but rather to establish that two methods are equal to one another. In equivalence testing, the null hypothesis is that the two groups are not equivalent to one another, and hence rejection of the null indicates that the two groups are equivalent. Therefore, as stated by Haller *et al*, there is a risk of accepting non-differentially expressed genes as suitable controls although they are not equivalently expressed [43]. Equivalence of expression between tumour and normal colorectal tissue was confirmed for all candidate EC genes using the equivalence test and a fold cut-off of 2. *DTX3, B2M, MRPL19 *and PPIA showed the minimum of variability in the confidence interval hence can be used for normalisation.

In their study to identify EC genes to monitor enterocyte differentiation and to compare normal and adenocarcinoma of the colon from microarray data, Dydensborg *et al *[5] recommended *RPLP0 *for normalising gene quantification in human intestinal epithelial cells and *B2M *for studying gene expression in human colon cancer. In addition, Blanquicett *et al *[44] analysed the extent of variability in gene expression between tumour and normal colorectal and liver tissues using two-tailed T tests. They showed that *18S, S9 *and *GUS *were the least variable genes in normal and metastatic liver specimens and were also appropriate for normal and tumour colorectal tissues. In the present study, we confirmed that more than one EC gene is required for optimal normalisation in colorectal tissue. We used clinico-pathologically diverse tissues to systematically evaluate normalisation of gene expression data in colorectal tissues. We also conducted equivalence testing to confirm the equality of expression of each EC gene. Thereby, the risks of incorrect rejection (type 1 error) and of false negativity (type 2 error) were minimised.

As stated above significant differences in target gene expression were noticed when using each of the EC genes and the combination of *PPIA *and *B2M*. Moreover, significant effect of EC on the magnitude of error associated with estimation of target gene expression was also determined in this study (figure 6). Our results were further confirmed by post hoc testing of individual levels of EC gene expression (Additional files 1). Reduction in the magnitude of error achieved using the combination of *PPIA *and *B2M *in comparison to using individual EC genes alone, further indicates that using two EC genes to normalise real-time data achieves greater accuracy in the determination of gene expression levels.

## Conclusions

The findings reported in this study confirm that use of two EC genes to normalise RQ-PCR data resulted in superior accuracy in the quantification of gene expression in colorectal tissue. The combined use of *B2M *and *PPIA *was validated as the optimal pair of EC genes with which to estimate the expression of all four target genes in colorectal cancer tissue. Although these ECs may not be ideal in other tissue types, the approach described herein could serve as a template to identify valid ECs in other tissue types.

## Methods

### Tissue Samples

A study group of 64 biopsies of human colon tissue samples was gathered from consenting patients at the time of primary curative surgical resection at Galway University Hospital, Ireland. The cohort comprised of 30 colorectal tumour specimens and 34 and tumour-associated normal (TAN) tissues. Following excision, all samples were subject to histopathological review prior immediate snap-freezing in liquid nitrogen and archival at -80°C until further use. Concomitant clinicopathological data on patients and specimens was obtained from the Department of Surgery Biobank, NUI Galway as detailed in Table 4. Ethical approval for this study was granted by the Clinical Research Ethics Committee, Galway University Hospitals.

**Table 4:** Candidate endogenous control (EC) genes and their PCR amplification efficiencies (E)

EC	Function	Chromosomal Location	Amplicon Size (bp)	Assay Identifier*	E (%)
B2M	Defence/immunity	15q21-22.2	64	Hs00187842_m1	101.8

GAPDH	Oxidoreductase, dehydrogenase	12p13	122	Hs99999905_m1	99.8

PPIA	Isomerase	7p13	98	Hs99999904_m1	96.6

HPRT	Glycosyl transferase	Xq26.1	100	Hs99999909_m1	97.9

MRPL19	Protein biosynthesis	2q11.1-11.2	72	Hs00608519_m1	102.2

ACTB	Cytoskeletal structure	7p15-12	171	Hs99999903_m1	95.2

DTX3	Signals transduction	12q13.3	64	Hs00400987_m1	99.1

SLC25A23	Mitochondrial carrier	19p13.3	86	Hs00225469_m1	97.8

CHRNB4	Nicotinic receptor	15q24	75	Hs00609523_m1	103.6

RTDR1	Aminopeptidase transport	22q11.2	112	Hs00205353_m1	UD

HCRT	Homeostatic regulator	12q21	101	Hs00533664_m1	UD

APOC4	Apo-lipoprotein	19q.2	144	Hs00155791_m1	UD

KRTAP12-3	Acetylgalactoa-minyltransferase	3q25	83	Hs01651247_s1	UD

* Applied Biosystems TaqMan^® ^gene expression assay ID

UD: undetermined

### RNA Extraction and Analysis

Tissue samples (50-100 mg) were homogenised using a hand-held homogenizer (Polytron^® ^PT1600E, Kinematica AG, Littau-Luzem, Switzerland) in 1-2 ml of QIAzol reagent (Qiagen, Crawley, UK). To minimise variation in sample processing, tumour and TAN samples were homogenised separately, but on the same day. RNA was extracted as previously described (Davoren *et al*) using the RNeasy^® ^Plus Mini Kit and RNeasy MinElute^® ^cleanup kit (Qiagen, Crawley, UK) according to the manufacturer's instructions. Briefly, large (> 200 nt) and small RNA (< 200 nt) fractions were isolated separately. For this study, only large RNA was utilised for further analysis. RNA was eluted in 60 μl volumes and stored at -80°C.

RNA concentration and purity was assessed in duplicate samples using a using a NanoDrop™ ND-1000 Spectrophotometer (Thermo Fisher Scientific, USA). RNA integrity was evaluated using the RNA 6000 Nano Chip Kit (Series II) and the Agilent 2100 Bioanalyzer System (Agilent technologies, Palo Alto, CA, USA). An RNA integrity number (RIN) was generated for each sample using the Agilent 2100 Expert Software (Version B.02.03) based on the ratio of ribosomal bands and also the presence or absence of degradation products on the electrophoretic and gel-like images. A threshold value of RIN ≥ 7 was applied and RNA purity was verified by an average A260/A280 ratio of 1.98 (range 1.97-2.01) and A260/A230 ration of 1.7 (range 1.5-1.83).

### Candidate Endogenous Control Genes

Based on literature search six commonly used candidate endogenous control genes were selected for analysis: *ACTB, GAPDH, HPRT, B2M, PPIA *and *MRPL19*. An additional panel of seven genes: *HCRT, SLC25A23, DTX3, APOC4, RTDR1, KRTAP12-3 and CHRNB4*, was also selected for analysis (Table 2). To our knowledge all genes have independent cellular functions and were assumed not to be co-regulated.

### cDNA Synthesis and RQ-PCR

First strand cDNA was synthesised using Superscript™ III reverse transcriptase (Invitrogen Life technologies, Paisley, UK) and random primers (N9; 1 μg, MWG Biotech, AG, Ebersberg, Germany). Negative control samples were included in each set of reactions. Reactions were incubated at 25°C for 5 minutes followed by 50°C for 1 hour and final denaturation at 72°C for 15 minutes. Samples were subsequently diluted to 50 μL in nuclease-free water and stored at -20°C. The expression of each EC gene was analysed by RQ-PCR using TaqMan^® ^gene expression assays using a 7900HT instrument (Applied Biosystems, Foster city, USA). All reactions were performed in 20 μL reactions, in triplicate within the same PCR run. Negative controls were included for each gene target under assay. On each plate, an interassay control was included to account for any variations between runs. For each well 2 μl of cDNA from each sample was added to 18 μl of PCR reaction mix which consisted of 10× TaqMan^® ^universal master mix, No AmpErase UNG, 7× nuclease free water and 1× gene expression assay primer-probe mix (Applied Biosystems, Foster city, USA). The PCR reactions were initiated with a 10 minute incubation at 95°C followed by 40 cycles of 95°C for 15 seconds and 60°C for 60 seconds, in accordance with the manufacturer's recommendations.

### PCR Amplification Efficiency

Amplification efficiencies for each EC gene assay were calculated applying the formula E = (10-1/slope - 1) × 100, using the slope of the plot of Ct versus log input of cDNA (10-fold dilution series). A threshold of 10% above and below 100% efficiency was applied. PCR amplification efficiency for each candidate EC gene is shown in table 2.

### Data Analysis

Cycle threshold (C_t_) is defined as the PCR cycle number at which the fluorescence generated from amplification of the target gene within a sample increases to a threshold value of 10 times the standard deviation of the base line emission and is inversely proportionate to the starting amount of the target cDNA. QBasePlus was used for calculation of *PDCD4 *expression relative to each of the EC genes. It applies ΔΔC_t _method was used where ΔΔCt = (C_t _target gene, test sample - C_t _endogenous control, test sample) - (C_t _target gene, calibrator sample - C_t _endogenous control, calibrator sample). Relative quantities were corrected for efficiency of amplification and fold change in gene expression between groups was calculated as E-ΔΔCt ± s.e.m. Where more than one endogenous control are used, fold change estimates were calculated using the geometric mean of EC quantities relative to the calibrator sample which could be the minimum, maximum or a named sample or an average.

Stability of the EC genes expression was evaluated with two freely available statistical models, geNorm and NormFinder. It is further validated with qBasePlus. Statistical analysis was carried out with Minitab^® ^15 (Minitab Ltd, Coventry, UK). Anderson-Darling normality test was applied and parametric tests were used where appropriate. The equivalence test was used to assess the equivalently of expression of the candidate genes between tumour and normal tissues. One-way ANOVA, two-sample t-test, Levene's test and Spearman and Pearson correlations were used to determine association and comparisons between groups. P values < 0.05 were considered statistically significant.

## Authors' contributions

EAHK performed the experiments, was responsible for data analyses and drafted the manuscript. KHC contributed to sample preparation from clinical samples and collation of clinicopathological data. NM conceived, designed and supervised experimental work and manuscript editing. JN contributed to statistical analysis of clinical data and drafting of the manuscript. MJK contributed throughout the experiment, critically reviewed the manuscript and participated clinically. All authors read and approved the final manuscript.

## Supplementary Material

Additional file 1 **Table 1 Supplementary data**. Post hoc testing of individual levels of EC gene expression. Click here for file
