# Supplementary material for: Identification of endogenous control genes for normalisation of real-time quantitative PCR data in colorectal cancer
Source: BMC Mol Biol. 2010 Feb 1;11:12. doi: 10.1186/1471-2199-11-12 (PMC2825202; doi:10.1186/1471-2199-11-12)
Supplement: Additional file 1 — Table 1 Supplementary data. Post hoc testing of individual levels of EC gene expression. [file 1471-2199-11-12-S1.PPT]

## Slide 1
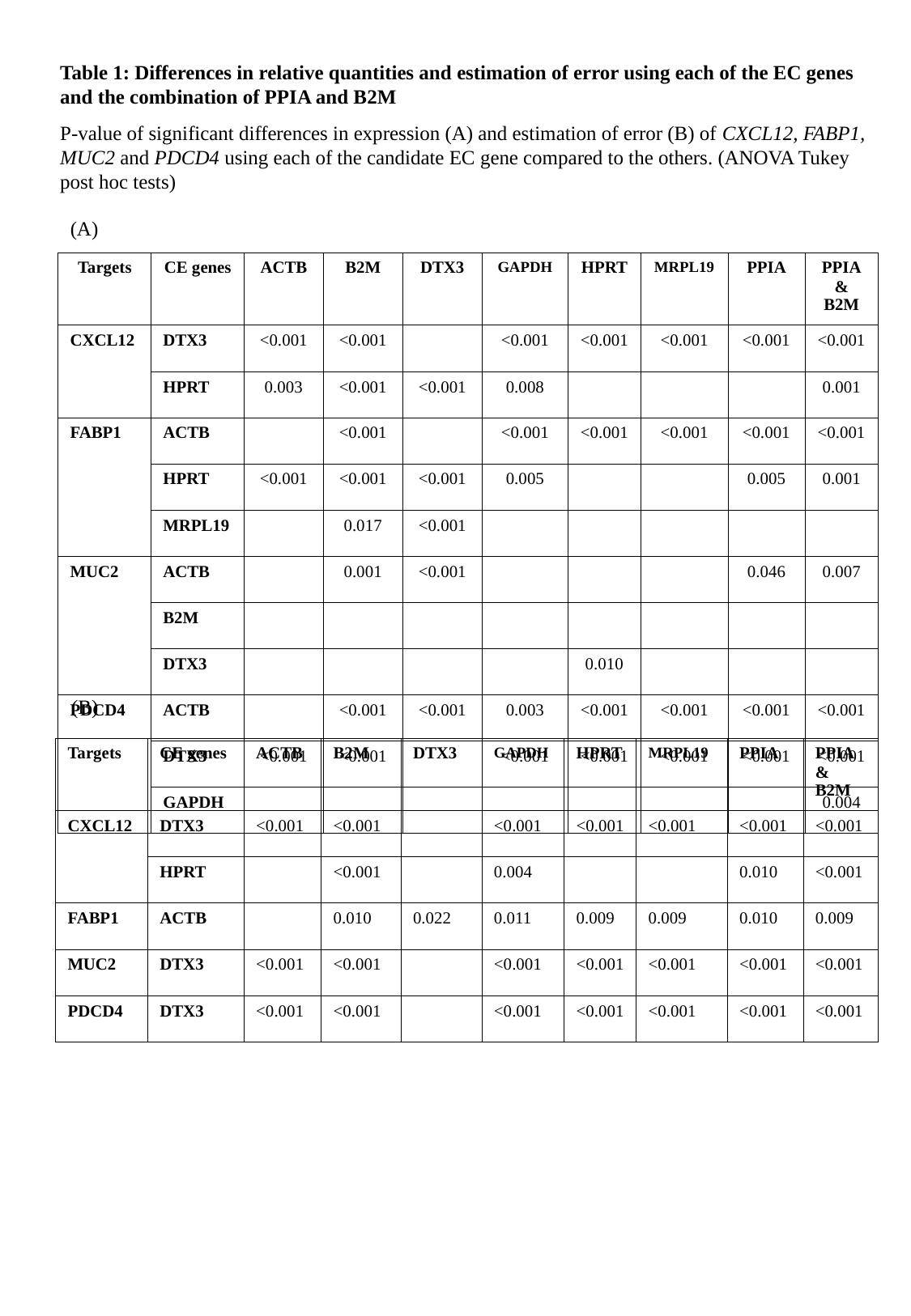

Table 1: Differences in relative quantities and estimation of error using each of the EC genes and the combination of PPIA and B2M
P-value of significant differences in expression (A) and estimation of error (B) of CXCL12, FABP1, MUC2 and PDCD4 using each of the candidate EC gene compared to the others. (ANOVA Tukey post hoc tests)
(A)
| Targets | CE genes | ACTB | B2M | DTX3 | GAPDH | HPRT | MRPL19 | PPIA | PPIA & B2M |
| --- | --- | --- | --- | --- | --- | --- | --- | --- | --- |
| CXCL12 | DTX3 | <0.001 | <0.001 | | <0.001 | <0.001 | <0.001 | <0.001 | <0.001 |
| | HPRT | 0.003 | <0.001 | <0.001 | 0.008 | | | | 0.001 |
| FABP1 | ACTB | | <0.001 | | <0.001 | <0.001 | <0.001 | <0.001 | <0.001 |
| | HPRT | <0.001 | <0.001 | <0.001 | 0.005 | | | 0.005 | 0.001 |
| | MRPL19 | | 0.017 | <0.001 | | | | | |
| MUC2 | ACTB | | 0.001 | <0.001 | | | | 0.046 | 0.007 |
| | B2M | | | | | | | | |
| | DTX3 | | | | | 0.010 | | | |
| PDCD4 | ACTB | | <0.001 | <0.001 | 0.003 | <0.001 | <0.001 | <0.001 | <0.001 |
| | DTX3 | <0.001 | <0.001 | | <0.001 | <0.001 | <0.001 | <0.001 | <0.001 |
| | GAPDH | | | | | | | | 0.004 |
(B)
| Targets | CE genes | ACTB | B2M | DTX3 | GAPDH | HPRT | MRPL19 | PPIA | PPIA & B2M |
| --- | --- | --- | --- | --- | --- | --- | --- | --- | --- |
| CXCL12 | DTX3 | <0.001 | <0.001 | | <0.001 | <0.001 | <0.001 | <0.001 | <0.001 |
| | HPRT | | <0.001 | | 0.004 | | | 0.010 | <0.001 |
| FABP1 | ACTB | | 0.010 | 0.022 | 0.011 | 0.009 | 0.009 | 0.010 | 0.009 |
| MUC2 | DTX3 | <0.001 | <0.001 | | <0.001 | <0.001 | <0.001 | <0.001 | <0.001 |
| PDCD4 | DTX3 | <0.001 | <0.001 | | <0.001 | <0.001 | <0.001 | <0.001 | <0.001 |
